# Reinforcement of cell-mediated immunity driven by tumor-associated Epstein-Barr virus (EBV)-specific T cells during targeted B-cell therapy with rituximab

**DOI:** 10.3389/fimmu.2023.878953

**Published:** 2023-03-24

**Authors:** Sabine Tischer-Zimmermann, Agnes Bonifacius, Maria Michela Santamorena, Philip Mausberg, Sven Stoll, Marius Döring, Ulrich Kalinke, Rainer Blasczyk, Britta Maecker-Kolhoff, Britta Eiz-Vesper

**Affiliations:** ^1^ Institute of Transfusion Medicine and Transplant Engineering, Hannover Medical School, Hannover, Germany; ^2^ Institute for Experimental Infection Research, TWINCORE, Centre for Experimental and Clinical Infection Research, A Joint Venture Between The Helmholtz Centre for Infection Research and Hannover Medical School, Hannover, Germany; ^3^ Department of Paediatric Hematology and Oncology, Hannover Medical School, Hannover, Germany; ^4^ German Center for Infection Research (DZIF), Site Hannover-Braunschweig, Hannover, Germany

**Keywords:** rituximab, cross-presentation, Epstein-Barr virus (EBV), cytotoxic T cells (CTL), EBV^+^ B-LCLs

## Abstract

**Introduction:**

In immunocompromised patients, Epstein-Barr virus (EBV) infection or reactivation is associated with increased morbidity and mortality, including the development of B-cell lymphomas. The first-line treatment consists of reduction of immunosuppression and administration of rituximab (anti-CD20 antibody). Furthermore, the presence of EBV-specific T cells against latent EBV proteins is crucial for the control of EBV-associated diseases. Therefore, in addition to effective treatment strategies, appropriate monitoring of T cells of high-risk patients is of great importance for improving clinical outcome. In this study, we hypothesized that rituximab-mediated lysis of malignant EBV-infected B cells leads to the release and presentation of EBV-associated antigens and results in an augmentation of EBV-specific effector memory T-cell responses.

**Methods:**

EBV-infected B lymphoblastoid cell lines (B-LCLs) were used as a model for EBV-associated lymphomas, which are capable of expressing latency stage II and III EBV proteins present in all known EBV-positive malignant cells. Rituximab was administered to obtain cell lysates containing EBV antigens (AC^EBV^). Efficiency of cross-presentation of EBV-antigen by B-LCLs compared to cross-presentation by professional antigen presenting cells (APCs) such as dendritic cells (DCs) and B cells was investigated by *in vitro* T-cell immunoassays. Deep T-cell profiling of the tumor-reactive EBV-specific T cells in terms of activation, exhaustion, target cell killing, and cytokine profile was performed, assessing the expression of T-cell differentiation and activation markers as well as regulatory and cytotoxic molecules by interferon-γ (IFN-γ) EliSpot assay, multicolor flow cytometry, and multiplex analyses.

**Results:**

By inhibiting parts of the cross-presentation pathway, B-LCLs were shown to cross-present obtained exogenous AC^EBV^-derived antigens mainly through major histocompatibility complex (MHC) class I molecules. This mechanism is comparable to that for DCs and B cells and resulted in a strong EBV-specific CD8^+^ cytotoxic T-cell response. Stimulation with AC^EBV^-loaded APCs also led to the activation of CD4^+^ T helper cells, suggesting that longer peptide fragments are processed *via* the classical MHC class II pathway. In addition, B-LCLs were also found to be able to take up exogenous antigens from surrounding cells by endocytosis leading to induction of EBV-specific T-cell responses although to a much lesser extent than cross-presentation of AC^EBV^-derived antigens. Increased expression of activation markers CD25, CD71 and CD137 were detected on EBV-specific T cells stimulated with AC^EBV^-loaded APCs, which showed high proliferative and cytotoxic capacity as indicated by enhanced EBV-specific frequencies and increased secretion levels of cytotoxic effector molecules (e.g. IFN-γ, granzyme B, perforin, and granulysin). Expression of the regulatory proteins PD-1 and Tim-3 was induced but had no negative impact on effector T-cell functions.

**Conclusion:**

In this study, we showed for the first time that rituximab-mediated lysis of EBV-infected tumor cells can efficiently boost EBV-specific endogenous effector memory T-cell responses through cross-presentation of EBV-derived antigens. This promotes the restoration of antiviral cellular immunity and presents an efficient mechanism to improve the treatment of CD20^+^ EBV-associated malignancies. This effect is also conceivable for other therapeutic antibodies or even for therapeutically applied unmodified or genetically modified T cells, which lead to the release of tumor antigens after specific cell lysis.

## Introduction

More than 90% of adults and 50% of children worldwide are infected with the human herpesvirus 4, also known as Epstein-Barr virus (EBV) (1–3). EBV exploits several powerful strategies to evade host immune responses and following resolution of primary infection, EBV establishes a lifelong latency in memory B cells, which is usually clinically unremarkable. EBV latency is discriminated into four latency stages, which can be distinguished based on the differential expression of a small number of EBV proteins, e.g. Epstein-Barr nuclear antigen 1 (EBNA1), EBNA2, EBNA3, and latent membrane protein (LMP) 2 (4).

Patients with congenital or acquired immune deficiency are at high risk to develop EBV-associated diseases; among those are a wide range of B-cell lymphomas, including Burkitt’s lymphoma, Hodgkin’s disease and post-transplant lymphoproliferative disorders (PTLD) (5–7). PTLD is an often fatal complication after solid organ (SOT) or hematopoietic stem cell transplantation (HSCT) (8). In HSCT and pediatric SOT, approximately 60-80% of PTLDs are EBV-associated (8, 9). More than 90% of EBV-seronegative patients developing an EBV primary infection with up to 33% of these primary infections develop into true PTLD (9). Delayed EBV-specific T-cell reconstitution in immunocompromised patients contributes to PTLD development during the early post-transplant phase (10). Assessment of EBV DNA load and appropriate T-cell monitoring for early identification of patients at high risk as well as effective treatment strategies are of great importance to improve clinical outcome in these patients.

Over the years, several strategies have evolved to treat EBV-associated malignancies. Reduction of immunosuppression (RI) as tolerated is a cornerstone to restore EBV-directed immunity; however, it must be balanced against the risk of graft-versus-host-disease (GvHD) or organ graft rejection (5, 11). Treatment with rituximab, a chimeric monoclonal antibody (mAb), with or without  chemotherapy is currently accepted as the first-line choice for preemptive intervention and targeted treatment (12) in a broad variety of CD20^+^ B-cell malignancies, including EBV-positive lymphomas. For EBV-associated PTLD, rituximab treatment resulted in complete remission in more than 60% of patients (12–14). In patients failing on rituximab treatment, adoptive transfer of EBV-specific T cells restricted against immunodominant antigens derived from latently expressed EBV proteins represents a promising alternative (15). Adoptive transfer of EBV-specific T cells is well tolerated and highly effective in treating EBV-associated PTLD in the HSCT and SOT setting, providing long-term antiviral immunity and resulting in a complete remission rate of up to 70% (5, 16, 17). These findings demonstrate that a combination of therapies consisting of (a) treatment with rituximab for targeted lysis of CD20^+^ EBV-infected tumor cells and (b) adoptive T-cell transfer to eliminate EBV-infected tumor cells and restore cellular immunity can result in long-lasting control of EBV (18, 19).

Different mechanisms of actions have been discovered for rituximab efficacy, among them antibody-dependent cell-mediated (ADCC) and complement-dependent cytotoxicity (CDC), which will result in cell lysis and thus in release of cells and cell membrane components, including EBV-derived proteins and peptides. Professional antigen-presenting cells (APCs) such as dendritic cells (DCs) are able to efficiently present these cell-associated antigens as well as antigens from virus-infected apoptotic and necrotic cells *via* the major histocompatibility complex (MHC) class I molecules. These antigens are then recognized by antigen-specific T cells in the context of MHC. Moreover, *in vivo* DCs are capable to phagocytose dead cells carrying antigens and migrate to the lymph nodes where they can cross-present these cell-associated antigens to CD8^+^ cytotoxic T lymphocytes (CTLs) by MHC class I (20). Cross-presentation plays an important role in the activation of cytotoxic antigen-specific memory T cells against viruses and tumors (21). The vacuolar and the cytosolic pathways are the two main models describing the mechanism of antigens cross-presentation by MHC class I molecules (21, 22). In addition to their essential role in the humoral immune response as antibody-secreting cells and by generating antibody-mediated memory responses, B cells are also capable of acting as professional APCs and thus play an important role in immune regulation and shaping of the evolving adaptive immune response (23). B cells are able to present antigens directly or by cross-presentation with enhanced capacity for antigen presentation in response to certain stimuli, comparable to that of DCs (24). Thus, they activate naive CD8^+^ T cells and promote robust CD4^+^ and CD8^+^ T-cell memory responses, while also playing a dominant role in recall responses (24–26).

The aim of this study was to investigate whether tumor-reactive EBV-specific memory T-cell responses can be enhanced by the presentation of EBV-associated antigens derived from rituximab-treated EBV-infected malignant B cells, which may provide an essential mechanism for the effective elimination of residual malignant cells *in vivo*. EBV-immortalized CD20^+^ B-cells (B-LCLs) were used as a suitable *in vitro* model for studying immune responses against EBV-associated lymphomas because they express latency states II and III - proteins, as do all known EBV-positive malignant cells (3, 4). Here we showed for the first time that rituximab-mediated lysis of EBV-infected tumor cells can efficiently enhance EBV-specific endogenous effector memory T cells responses through cross-presentation of EBV-derived antigens, thereby promoting restoration of antiviral cellular immunity. The results will contribute to better understand the complexity of rituximab therapy in malignant diseases with (a) direct effect (elimination of malignant B cells) and (b) indirect effect (activation and proliferation of endogenous EBV-specific T cells and other immune cells like macrophages, natural killer (NK) cells, NKT cells and regulatory T cells) and to advance the establishment of individualized and personalized therapies in the future.

## Materials and methods

### Isolation of PBMCs, monocytes, T cells and B cells

All experiments were performed with residual blood samples from platelet apheresis disposable kits used for routine platelet collection from EBV-seropositive or -seronegative healthy donors of the Hannover Medical School (MHH) Institute of Transfusion Medicine and Transplant Engineering. Informed consent was obtained from all donors following approval by the Ethics Committee of MHH (ethical number: 3639-2017, 2744-2015). All donors were pretested for EBV serostatus as described previously using commercially available IgG Western blot (27, 28). Peripheral blood mononuclear cells (PBMCs) were isolated using discontinuous gradient centrifugation. Untouched CD14^+^ monocytes and CD3^+^ T cells were enriched by magnetic cell sorting (MACS) using the Pan Monocyte and the Pan T cell Isolation Kit (Miltenyi Biotec, Bergisch Gladbach, Germany) according to the manufacturer’s instructions. CD20^+^ B cells were isolated using CD20 microbeads for positive selection (Miltenyi Biotec) and analyzed for purity by flow cytometry (**Supplemental Figure 1A**). Either fresh or frozen CD3^+^ T cells were used for immunoassays. For cryopreservation, T cells were resuspended in freezing medium (RPMI1640 (Lonza, Verviers, Belgium) + 20% AB serum (C.C.pro, Oberdorla, Germany) + 10% DMSO (Sigma-Aldrich, St Louis, MO) and stored at - 80°C until use.

### Generation of EBV-immortalized B lymphoblastoid cell lines

EBV-immortalized B lymphoblastoid cell lines (B-LCLs) were generated from PBMCs using established protocols (29). Briefly, 2 x 10^6^/ml PBMCs were resuspended in transformation medium consisting of RPMI1640 + 10% FBS (Lonza) and 200 ng/ml cyclosporine A (Norvatis, Nureberg, Germany) and seeded into a T25 flask (TPP, Trasadingen, Switzerland). Cells were infected with EBV strain B95-8 at 37°C and medium was changed once or twice per week. The EBV-infected cells were microscopically monitored with respect to formation of rosette-like B-LCL clusters and further examined for CD20 expression by flow cytometry (**Supplemental Figure 1B**). After three to four weeks, the generated B-LCLs showed the typical cell aggregation and served as both antigen source to generate the EBV antigen-containing cocktail (AC^EBV^) and APCs to study cross-presentation.

### *In vitro* differentiation of moDCs

CD14^+^ monocytes were seeded in DC culture medium (DC-CM, RPMI1640 + 2% AB serum; C.C.pro, Oberdorla, Germany) at a concentration of 1 x 10^6^ cells/ml in a 24-well flat bottom plate (Sarstedt, Nuembrecht, Germany). After one day of incubation at 37°C and 5% CO_2_, the complete supernatant was collected and stored as monocyte-conditioned medium at -20°C. The monocytes were resuspended in fresh DC-CM supplemented with 800 U/mL human granulocyte-macrophage colony-stimulating factor (GM-CSF, PeproTech, London, UK) and 500 U/ml recombinant human Interleukin-4 (rhIL-4; PeproTech) and incubated for five days. The nonadherent cells (immature DCs, imDCs) were collected, transferred to a new 24-well flat bottom plate and cultured in DC-CM supplemented with monocyte-conditioned medium and 20 ng/ml lipopolysaccharide (LPS, Sigma-Aldrich) for one further day to induce DC maturation. The differentiation of monocytes into moDCs was assessed by flow cytometry during the time of cultivation (**Supplemental Figure 1C**).

### Analysis of rituximab-mediated lysis and generation of antigen-containing cocktails AC^EBV^ and AC^ø^

Rituximab-mediated cell lysis was analyzed using B-LCLs and B cells from healthy EBV-seropositive and seronegative donors. Expression of CD20 on B-LCLs was determined by flow cytometry and only cells with a fraction of > 80% of CD20^+^ cells were used for further experiments. Cells were resuspended in serum-free RPMI1640 at a density of 2.5 x 10^5^ cells/well, supplemented with rituximab (Roche, Basel, Switzerland) at a final concentration of 0-1 mg/ml and incubated in a 48-well flat bottom plate (500 µl/well, Sarstedt) at 37°C and 5% CO_2_ for up to six days. Viability and total cell numbers were determined by light microscopy using trypan blue as well as by life imaging (Etaluma 500 Microscope, Etaluma, Carlsbad, CA). As an EBV-uninfected control, B cells were treated with rituximab to generate the AC^ø^. For the generation of AC^EBV^ and AC^ø^, 2.5 x 10^5^ B-LCLs or B cells, respectively, were incubated with 1 mg/ml rituximab in serum-free RPMI1640 for three days. The resulting cell culture media, including cells and cell debris, were collected without further washing steps to avoid loss of antigens. The concentration of generated ACs was defined according to the number of cells treated with rituximab (2.5 x 10^5^ cells).

### Verification of the presence of EBV-derived proteins in the AC^EBV^

To verify the presence of EBV proteins in the generated AC^EBV^, western blotting for the latent EBV proteins EBNA1 and LMP2A was performed. The respective origin B-LCLs and Raji cells (EBV-positive Burkitt’s lymphoma-derived cell line) were used as positive control, while HEK293T (EBV-negative), B cells and AC^ø^ served as negative control. Cell lysates were prepared from cell pellets using RIPA buffer (ThermoFisher Scientific, Waltham MA) supplemented with the protease inhibitor Mix M (SERVA, Heidelberg, Germany) and were cleared from cell debris by centrifugation. The generated ACs were used without washing. The samples were analyzed on 12% Bis/Tris NuPAGE gels (Invitrogen, Waltham, MA) under reducing conditions and transferred to PVDF membrane (0.2 µm; Bio-Rad, Hercules, CA). After blocking in 5% low-fat dry milk solution in TBS-T (0.05% Tween-20, Sigma-Aldrich, St. Louis, MS) the membranes were probed with either Horseradish Peroxidase (HRP)-conjugated anti-EBNA1 (1:2000; clone 1EB12, Santa Cruz Biotechnology, Dallas, Texas), anti-LMP2A (1:200; clone 15F9, Santa Cruz Biotechnology) or Glyceraldehyde-3-phosphate dehydrogenase (GAPDH) (1:200; clone 0411, Santa Cruz Biotechnology) antibody. The detection of GAPDH served as a loading control. After two hours incubation at room temperature, the immunoblots were probed with a polyclonal HRP-coupled secondary antibody (1:10000; Dako, Glostrup, Denmark) for one hour. Peroxidase activity was detected using chemiluminescence (Clarity Max Western ECL Substrate; Biorad, Hercules, California) according to manufacturer’s instructions.

### Analysis of TAP-dependent peptide translocation in B-LCLs

To investigate whether B-LCLs are able to take up exogenous antigens and effectively present them *via* MHC class I molecules, a transporter associated with antigen processing (TAP) -dependent peptide translocation assay was performed as previously described (30). Briefly, 2 × 10^5^ B-LCLs were resuspended in translocation buffer (PBS supplemented with 10 mM MgCl_2_, Sigma-Aldrich) and semipermeabilized using 0.25 μg/ml streptolysin O (SLO, Abcam, Cambridge, UK). 10 nM of the TAP-dependent human histone H3-derived reporter epitope RRYQNSTC^(F)^L labelled with fluorescein (NST-F, 50 µl total, Syro, MultiSynTech, Bochum, Germany) and 10 mM ATP or ADP (Sigma-Aldrich) were added and cells were incubated for 15 min at 37 °C. The reaction was stopped by addition of 150 μl PBS supplemented with 20 mM EDTA (Sigma-Aldrich), followed by analysis using flow cytometry (FACS LSRII, BD Biosciences). Samples were analysed by FlowJo 7.6.5 software reporting the fluorescein channel mean fluorescence intensity (MFI).

### Determination of EBV-specific memory T cells by IFN-γ EliSpot assay

Detection of EBV-specific IFN-γ-producing T cells was performed by target cell-dependent IFN-γ EliSpot assay as previously described (31) by using either B-LCLs, B cells or moDCs (APCs) as target cells. The APCs were loaded with AC^EBV^ or AC^ø^ in a 3:1 loading ratio (AC:APC) for 16 hours. As a positive control, APCs were loaded with EBV Consensus peptide pool (AC^Cons^; 1 µg per peptide/ml, Miltenyi Biotec). Subsequently, unloaded and loaded (AC^EBV^, AC^Cons^ or AC^ø^) target cells were resuspended in fresh T-cell culture medium consisting of RPMI1640 with 10% human AB serum (TCM) and added to a 96-well precoated EliSpot plate (Mikrogen Diagnostik, Neuried, Germany). Freshly isolated or cryopreserved autologous CD3^+^ T cells from EBV-seropositive and -seronegative donors served as effector cells. The CD3^+^ T cells were isolated or thawed, respectively, the day before and rested in TCM overnight. Effector to target (E:T) ratios of 10:1 and 4:1 were applied using 2.5 x 10^5^ T cells/well. Background response was determined by using CD3^+^ T cells and target cells alone. For positive control, CD3^+^ T cells were stimulated with 1 µg/mL staphylococcus enterotoxin B (SEB, Sigma-Aldrich). IFN-γ secretion was detected following 16 hours of incubation at 37°C and 5% CO_2_ using biotin-conjugated anti-human IFN-γ antibody (mAb 7-B6-1-biotin, Mabtech, Stockholm, Sweden) and streptavidin-alkaline phosphatase (Mabtech), revealed by 5-bromo-4-chloro-3-indolyl phosphate/nitroblue tetrazolium (BCIP/NBT Liquid Substrate, Sigma-Aldrich). Spots were counted using AID EliSpot 8.0 on an AID iSpot spectrum reader system (both from AID, Strassberg, Germany). All spot counts are mean values of duplicate wells and expressed as spot-forming unit per well (spw). Spot counted for CD3^+^ T cells or APCs alone were subtracted from those measured in T-cell co-cultures.

### Verification of AC^EBV^-derived antigen cross-presentation by B-LCLs

The ability of cross-presentation of AC^EBV^-derived antigens by B-LCLs was investigated by *in vitro* inhibition assays as described by Fonteneau et al. (22). Three inhibitors were used to block different parts of the cross-presentation pathway: (1) antigen uptake was blocked with dimethyl amiloride (DMA), an ion channel inhibitor; (2) Cytochalasin D (CCD) was used to block actin cytoskeleton distribution; and (3) Brefeldin A (BrfA) was used as inhibitor of protein trafficking in the endomembrane system. Briefly, B-LCLs were pre-treated with DMA (0 to 300 µM) or CCD (0 to 50 µM, both from Sigma-Aldrich) for 40 minutes at 37°C and subsequently incubated in presence of AC^EBV^ for six hours, followed by target-cell-dependent IFN-γ EliSpot assay as described above. To block the transport and presentation of AC^EBV^-derived antigens by MHC class I molecules in B-LCLs, cells were incubated with AC^EBV^ for four hours at 37°C, followed by incubation with increasing doses of BrfA (0 to 5 µg/ml, BioLegend, San Diego, CA) for a further six hours and subsequent target-cell-dependent IFN-γ EliSpot assay as described above. B-LCLs treated with inhibitors in absence of AC^EBV^ served as control.

### Antigen-specific stimulation to characterize T-cell response towards AC^EBV^

For *in vitro* stimulation of EBV-specific T cells, B-LCLs, B cells and moDCs (APCs) served as target cells and were loaded with AC^EBV^ in a 3:1 loading ratio or AC^Cons^ (positive control) overnight in serum-free RPMI1640. Unloaded APCs served as negative control. Target cells were irradiated (30 Gy) and incubated for seven days in 96-well round bottom plates (Sarstedt) with autologous CD3^+^ T cells at an E:T ratio of 10:1 in TCM containing 50 U/ml rhIL-2 (PeproTech). On days 2 and 5, rhIL- 2 (50 U/ml) was added. After seven days, cell culture supernatants were collected and stored at -20°C until further analysis. Cells were harvested and either used as effector cells in additional cytotoxicity assays, or – in case of T cells co-cultured with B-LCLs – restimulated. For the latter, B-LCLs were loaded with AC^EBV^ overnight and irradiated (30 Gy), followed by co-cultivation with the expanded T cells at an E:T ratio of 10:1 for additional seven days. TCM with 50 U/ml rhIL-2 was added on days 7, 9, and 12. On days 7 and 14, supernatants were collected and stored at - 20°C until further analyses, and cells were harvested and analyzed by flow cytometry.

### Analysis of the proliferative capacity of EBV-specific T cells and detection of HLA-restricted EBV-specific T cells by multimer staining

To determine the proliferative capacity of EBV-specific memory T cells, freshly isolated CD3^+^ T cells were labeled with CellTrace Violet (CTV) Proliferation Kit (2 mM, Thermo Fisher Scientifics, Waltham MA) according to the manufacturer’s instructions. CTV-labeled T cells were co-cultured with autologous AC^EBV^-loaded or unloaded (control) B-LCLs, B cells and moDCs at an E:T ratio of 10:1 in 96-well round bottom plates as described above. After seven days, T-cell proliferation was assessed by flow cytometry. Gating was carried out in accordance with the light scatter properties of lymphocytes with at least 50,000 events in the lymphocyte gate. The frequencies of proliferating CTV^low^ T cells co-cultured with the respective unloaded APCs were subtracted from the frequencies measured in co-cultures with AC^EBV^-loaded APCs.

To detect T cells against human leukocyte antigen (HLA) -restricted EBV epitopes, cultures with AC^EBV^-loaded or unloaded HLA-A*02:01-positive target cells (B-LCLs, B cells and moDCs) and autologous CD3^+^ T cells were analyzed by multimer staining using the HLA-A*02:01-restricted peptide from EBV protein LMP2A (CLGGLLTMV). The frequency of LMP2A-reactive CD8^+^ T cells (A02LMP2A^+^ T cells) was determined before and after antigen-specific stimulation (days 0/7) using the allophycocyanin (APC)-conjugated HLA-A^*^02:01/EBV_LMP2A (CLGGLLTMV)-specific dextramer (A02LMP2A; Immudex, Copenhagen, Denmark) according to the manufacturer’s instruction. An APC-conjugated HLA-A*02:01 nonsense dextramer (Immudex) served as negative control. Additionally, cells were stained for CD3 (fluorescein-isothiocyanate (FITC), clone UCHT1, BioLegend) and CD8 (AlexaFluor-700 (AF-700), clone SK1, BioLegend) and analyzed by flow cytometry. The gating was carried out in accordance with the light scatter properties of lymphocytes and at least 50,000 events in the lymphocyte gate were recorded. The frequencies of A02LMP2A^+^ T cells, forming a well-defined population were determined within CD3^+^CD8^+^ T cells. Frequencies measured using the nonsense dextramer were subtracted.

### Flow cytometry analysis to determine cell purity, phenotype, activation and exhaustion markers

The purity of isolated CD20^+^ B cells, CD14^+^ monocytes and CD3^+^ T cells was assessed by using anti-CD20 APC (clone 2H7), anti-CD14 brilliant violet (BV) 605 (clone M5E2) and anti-CD3 FITC (clone UCHT1) antibodies. For analysis of B-LCLs and CD20^+^ B cells, cells were stained with anti-CD19 APC (clone HlB19), anti-CD20 phycoerythrin/Cyanine 7 (PE-Cy7, clone 2H7), anti-HLA-ABC FITC (clone w6/32), and anti-HLA-DR APC-Cy7 (clone L243) antibodies. DC maturation was examined using anti-CD14 BV605 (clone M5E2), anti-CD83 APC (clone HB15e), anti-CD86 BV421 (clone BU63), anti-CD209 PE-Cy7 (clone 9E9A8), HLA-ABC PE (clone w6/32) and anti-HLA-DR APC-Cy7 antibodies. T-cell phenotype and exhaustion was analyzed by using anti-CD3 FITC (clone UCHT1), anti-CD4 peridinin chlorophyll (PerCP, clone SK3), anti-CD8 APC (clone SK1), anti-CD45RA BV605 (clone HI100), anti-CD62L BV510 (clone DREG-56), anti-Tim-3 BV421 (clone F38-2E2), anti-PD-1 PE (clone EH12.2H7), and anti-CTLA-4 PE-Cy7 (clone L3D10) antibodies. For analysis of T-cell activation, cell were stained with anti-CD3 FITC, anti-CD4 PerCP, anti-CD69 BV605 (clone FN50), anti-CD71 PE (clone CZ1G4), anti-CD25 PE-Cy7 (clone BC96), and anti-CD137 APC (clone 4B4-1) antibodies. All antibodies were purchased from BioLegend.

Cells were stained for 20 min at room temperature in the dark, washed with PBS supplemented with 0.1% human AB serum and analyzed by multicolor flow cytometry (FACSCanto 10c system, BD Biosciences, Heidelberg, Germany). Gates were set based on the forward scatter versus side scatter properties of leukocytes. At least 10,000 events were acquired in the leukocyte gate.

### Analysis of the cytotoxic potential of EBV-specific T cells by flow cytometry

T cells stimulated over seven days with AC^EBV^-loaded, AC^Cons^-loaded or unloaded B-LCLs, B-cell or moDCs were evaluated for their cytotoxic potential. For this purpose, they were seeded with CTV-stained autologous B-LCLs, unloaded PBMCs, or B cells in 96-well round bottom plates at E:T ratio of 1:1 and 5:1. Target cell viability was verified in the absence of T cells. After four hours, cells were washed, stained with 7-AAD, and analyzed by flow cytometry. The gating was carried out in accordance with the light scatter properties of the target cells (B-LCLs, PBMCs and B cells). With at least 10,000 events in the CTV^+^ target cell gate, the focus of the gating strategy was set on the 7-AAD^+^ cells in order to assess dead cells. Values obtained from co-cultures with PBMCs or B cells (EBV-uninfected controls) were subtracted.

### Detection of cytotoxic effector molecules in cell culture supernatants secreted by EBV-specific T cells

The collected cell culture supernatants were analyzed *via* LEGENDPlex™ assay using the Human CD8/NK Cell Kit (BioLegend) according to the manufacturer’s instructions. Samples were measured by flow cytometry.

### Data analysis

Data were analyzed using BD FACSDiva v8.0.1 (BD Biosciences), FlowJo™ v10.6.2 (FlowJoTM LLC, BD Biosciences), LEGENDPlex™ Software (v8.0), and Microsoft Excel 2016 (Microsoft Corporation, Redmond). Graphs and statistical analysis were generated using Prism Version 9.3.1 (GraphPad Software, San Diego, California). Statistical analysis was performed using non-parametric (paired: Wilcoxon; unpaired: Mann-Whitney test) and 2way ANOVA test followed by multiple comparison. Results are presented as means ± standard deviation (SD) and levels of significance were calculated and expressed as p-values (*p < 0.05, **p < 0.01; ***p < 0.001 ****p < 0.001).

## Results

### Antibody-mediated lysis proved more effective in B cells compared to B-LCLs as a tumor model

**Figure 1:**
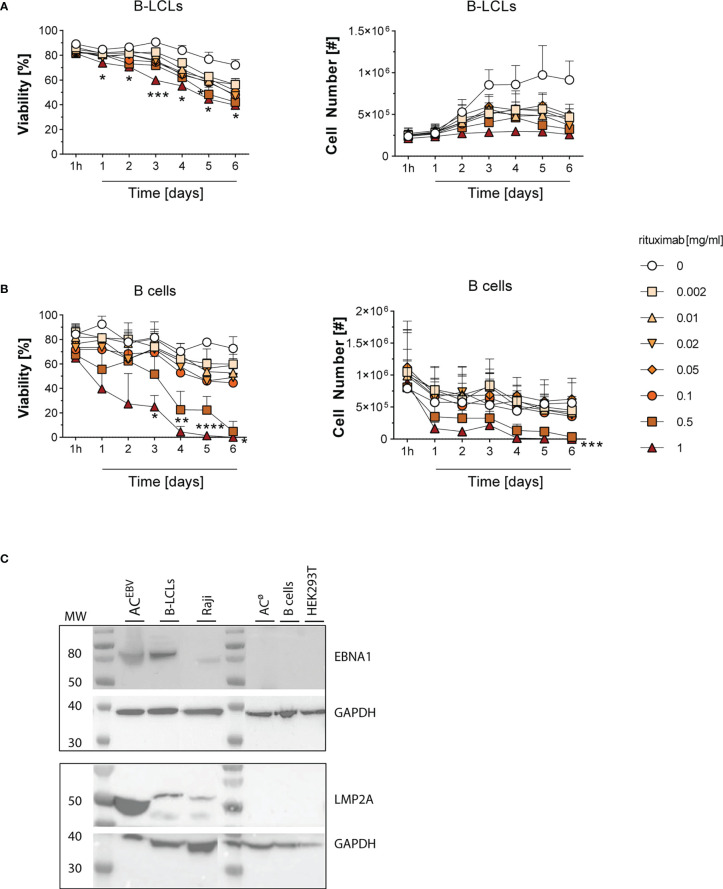
Rituximab-mediated cell lysis in B-LCLs and B cells and detection of EBV proteins in the B-LCLs lysate. Viability and total numbers of **(A)** EBV-immortalized B-lymphoblastoid cell lines (B-LCLs, n=3) and **(B)** isolated CD20^+^ B cells (n=3) from different healthy EBV-seropositive blood donors treated with rituximab at different concentrations (0-1 mg/ml) for one hour to six days. Results of three independent experiments for B-LCLs and B cells, respectively, are expressed as mean ± SD. Statistical significance was calculated using the 2way ANOVA test followed by multiple comparison (*p < 0.05, *pp < 0.01, ***p < 0.001, ****p< 0.0001). **(C)** Detection of EBV proteins EBNA1 and LMP2A in lysate of rituximab-treated B-LCLs (AC^EBV^, 1 mg/ml rituximab for three days) by western blot immunostaining. Cell lysates from the respective B-LCLs and Raji cells were used as positive control, whereas HEK293T, B cells and rituximab-treated B cells (AC^ø^) served as negative control. Glyceraldehyde-3-phosphate dehydrogenase (GAPDH) was used as loading control. MW - molecular weight.

To evaluate the efficacy of rituximab-induced cell lysis of B-LCLs and isolated CD20^+^ B cells (mean purity >90%, **Supplementary Figure 1A**), cells from six healthy donors were treated with increasing concentrations of rituximab (0.002 to 1 mg/ml) in serum-free TCM for up to six days (**Figure 1**). The mean viability of B-LCLs and B cells used in this study was 89% and 84%, respectively, and remained stable during six days of culture. With increasing rituximab concentration and over time, fewer viable cells were observed in both B-LCL and B-cell cultures, indicating concentration dependent rituximab-mediated cell lysis. Overall, B-LCLs were more resistant in a time- and concentration-dependent manner, and still showed mean viability of 39.7% after six days in the presence of 1 mg/ml rituximab, (**Figure 1A**). B cells were more sensitive to rituximab compared to B-LCLs, as their viability decreased rapidly at rituximab concentrations of 0.5 to 1 mg/ml and was below 5% after six days (**Figure 1B**). For preparation of AC^EBV^, to avoid spontaneous cell lysis due to a long cultivation time and to preserve the cell lytic effect of rituximab, the respective cells were incubated in presence of 1 mg/ml rituximab for three days. Furthermore, the presence of EBV-derived antigens in AC^EBV^ was determined by western blot immunostaining based on the detection of the latent EBV proteins EBNA1 and LMP2A (**Figure 1C**). EBNA1 and LMP2A proteins were detectable in AC^EBV^, the respective origin B-LCLs and Raji cells but not in AC^ø^, B cells and HEK293T. Overall, rituximab-mediated lysis of B-LCL and B cells as well as presence of latent EBV proteins in the generated AC^EBV^ was confirmed.

### B-LCLs are capable of TAP-dependent peptide translocation

**Figure 2:**
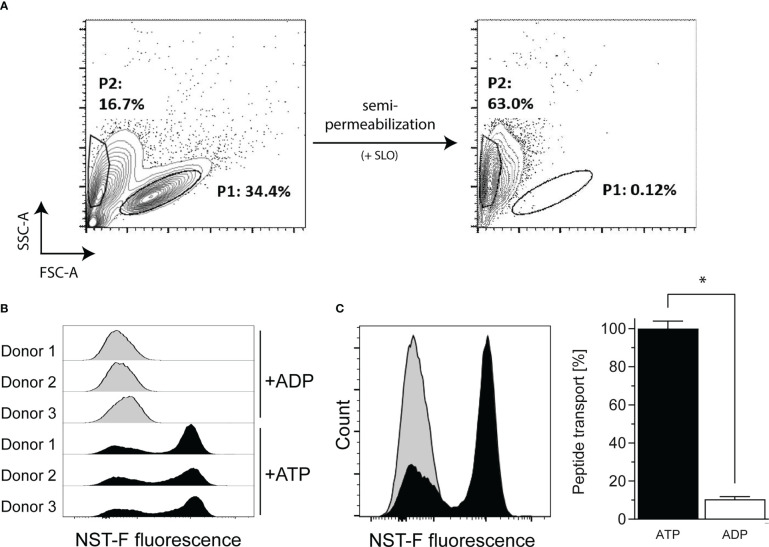
TAP-dependent peptide translocation assay in B-LCLs to monitor TAP-dependent antigen translocation by flow cytometry. B-LCLs resuspended in translocation buffer were **(A)** semipermeabilized with streptolysin O (SLO) and 10 nM fluorescein-labelled human histone H3-derived reporter peptide RRYQNSTC^(F)^L (NST-F) was added. The viable B-LCL population (P1) was shifted in the SSC/FSC scatter plot after semipermeabilization. Apoptotic cells without SLO treatment showed no background transport. Peptide translocation was carried out in the presence of ATP or ADP (10 mM each). ATP-dependent compartmentalization of NST-F shown as mean fluorescence intensity (MFI) of the fluorescein channel for ATP versus ADP sample in **(B)** histogram overlay of the single-cell events for all tested samples and as **(C)** histogram overlay for one representative result and as mean relative translocation rate (%, ATP: black bar versus ADP: white bar). Results of three independent experiments are expressed as mean ± SD (n=3). Statistical significance was calculated using the non-parametric Mann-Whitney test (*p < 0.05).

Up to now, it was unclear whether B-LCLs are able to effectively present exogenous antigens *via* MHC class I molecules similar to what is known for DCs and B cells. Therefore, in a flow cytometry-based *in vitro* assay, TAP-dependent peptide translocation was investigated (30, 32). Semipermeabilized B-LCLs were loaded with the fluorescently labeled peptide NST-F, which allowed monitoring of TAP-dependent intracellular compartmentalization (**Figure 2A**). NST-F was glycosylated to avoid the endoplasmic reticulum (ER)-associated degradation pathway followed by retrotranslocation (30). A low frequency of apoptotic cells was detected before semipermeabilization (P2, 16.7%, **Figure 2A**) and these cells were incapable of TAP-dependent NST-F peptide translocation in the presence of exogenous ATP, suggesting that there is no background transport by apoptotic cells (data not shown). Successful semipermeabilization of viable B-LCLs (P1) was confirmed by a shift in the forward scatter signal (FSC-A). Only in the presence of ATP, but not in the presence of ADP, the NST-F-mediated fluorescence signal was increased, with a maximal signal observed within ~15 min of incubation (**Figure 2B**). This is indicative of ATP-dependent translocation of NST-F. A constant peptide translocation background of 10.5%, which was evident in the ADP samples, can be attributed to nonspecific binding of NST-F to the semipermeabilized cells (**Figure 2C**). Taken together, these data demonstrate TAP activity and TAP-dependent peptide translocation in B-LCLs, which is comparable to that described for monocytes and B cells (32).

### AC^EBV^-derived antigens stimulate EBV-specific memory T cells

**Figure 3:**
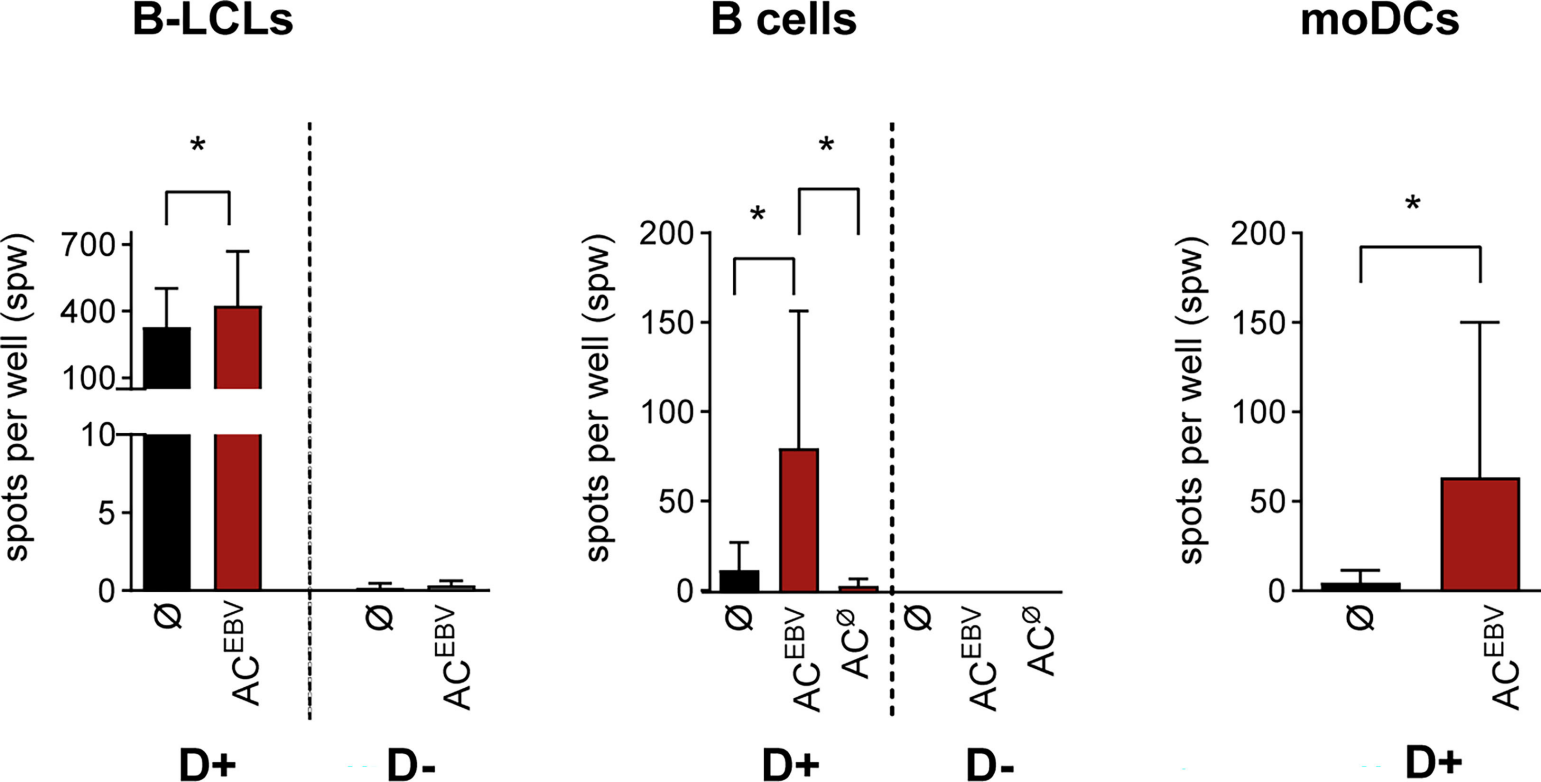
EBV-specific T-cell stimulation through AC^EBV^-derived antigens presented by human APCs. EBV-specific T-cell responses examined in EBV-seropositive (D+, n=7) and -seronegative (D-, n=3) donors by IFN-γ EliSpot assay using CD3^+^ T cells co-cultured with either autologous AC-loaded B-LCLs (effector to target (E:T) ratio 10:1, 2.5 x 10^5^ CD3^+^ T cells), B cells (E:T ratio 4:1, 2.5 x 10^5^ CD3^+^ T cells) or moDCs (E:T ratio 4:1, 1 x 10^5^ CD3^+^ T cells) as APCs (AC^EBV^/AC^ø^, red bars). T-cell co-cultures with unloaded APCs (Ø, black bars) in same cell numbers and ratios served as controls. All spot counts are mean values of duplicate wells and expressed as spot-forming unit per well (spw). Spot counted for CD3^+^ T cells or APCs alone were subtracted from those measured in T-cell co-cultures. Results of independent experiments are expressed as mean ± SD. Statistical significance was calculated using the non-parametric Wilcoxon test (*p<0.05). AC– antigen-containing cocktail derived from B-LCLs (AC^EBV^) or B cells (AC^Ø^) after rituximab treatment.

Using IFN-γ EliSpot assays, we determined whether antigens derived from rituximab treated B-LCLs (AC^EBV^) can stimulate EBV-specific CD3^+^ memory T cells by presentation through B-LCLs, B cells, or moDCs as APCs (**Figure 3**). In co-cultures of T cells from EBV-seropositive donors with AC^EBV^-loaded APCs, an increased frequency of IFN-γ-secreting cells, defined by the number of spots per well (spw), was observed compared with unloaded controls. In contrast, no T-cell response was observed when using T cells from an EBV-seronegative donor, indicating that the observed responses are memory T-cell responses. Because EBV-specific antigens are already presented on B-LCLs, a higher background of IFN-γ^+^ T-cell responses was observed when unloaded B-LCLs were used as APCs compared with B cells and moDCs. However, T-cell responses in co-cultures with AC^EBV^-loaded B-LCLs (mean 426 spw) were significantly higher than in unloaded B-LCLs (mean 328 spw, *p < 0.05). The use of AC^EBV^-loaded B cells or moDCs resulted in the stimulation of EBV-specific memory T cells with a lower background response compared with B-LCLs.

### B-LCLs can cross-present AC^EBV^-derived antigens *via* a common pathway involving uptake, processing, and presentation

**Figure 4:**
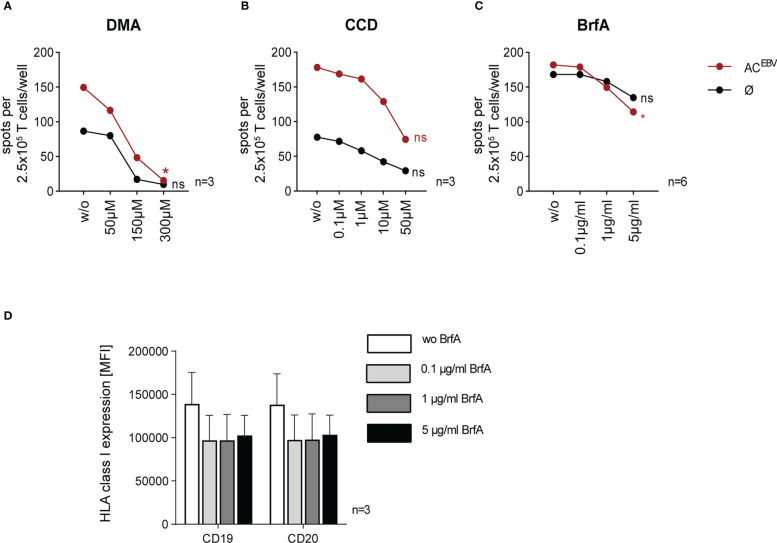
Reduction of EBV-specific T-cell responses by inhibiting parts of the cross-presentation pathway in AC^EBV^-loaded B-LCLs. Cross-presentation of AC^EBV^ by B-LCLs investigated by *in vitro* inhibition assays (22). B-LCLs pretreated with increasing concentrations of **(A)** dimethyl amiloride (DMA, 0 to 300 µM, n=3) and **(B)** cytochalasin D (CCD, 0 to 50 µM, n=3) before loading with the EBV antigen cocktail (AC^EBV^). Using **(C)** brefeldin A (BrfA, 0 to 5 µg/ml, n=6) cells were treated after AC^EBV^-loading. For control, with inhibitors treated unloaded B-LCLs (Ø) were used. Specific T-cell responses monitored by IFN-γ EliSpot assay using autologous 2.5 x 10^5^ CD3^+^ T cells as effector cells and inhibitor-treated B-LCLs at an E:T ratio of 10:1. **(D)** Mean fluorescence intensity (MFI, n=3) of HLA class I expression on CD19^+^ and CD20^+^ B-LCL in the presence of different BrfA concentrations. Results of independent experiments are expressed as mean ± SD. Statistical analysis was performed using the non-parametric Wilcoxon test (*p<0.05) and ns - not significant.

To verify whether EBV-specific memory T-cell responses were the results of cross-presentation of AC^EBV^-derived antigens, B-LCLs were treated with inhibitors of the antigen presentation pathways: DMA and CCD inhibit the uptake of exogenous antigens while BrfA inhibits protein transport to the cell surface, including exogenous and endogenous antigens. Inhibitor treatment did not affect cell viability (data not shown). In accordance with the data shown in **Figure 3**, co-cultures with unloaded B-LCLs not treated with inhibitors resulted in detectable EBV-specific memory T-cell responses in EBV-seropositive donors, which were increased in co-cultures with AC^EBV^-loaded B-LCLs (**Figures 4A–C**). B-LCLs preincubated with increasing concentrations of DMA prior to AC^EBV^ loading resulted in up to 10-fold decreased specific memory T-cell responses (w/o: 150 spw; 300 µM DMA: 15 spw; **Figure 4A**). As shown in **Figure 4B**, EBV-specific T-cell responses towards AC^EBV^-loaded B-LCLs were reduced by 2.4-fold after pretreatment with 50 µM CCD (w/o: 178 spw; 50 µM CCD: 75 spw). Interestingly, EBV-specific T-cell responses were also reduced when unloaded B-LCLs were preincubated with DMA or CCD, indicating uptake of exogenous antigens by unloaded B-LCLs, e.g. from surrounding apoptotic B-LCLs. When pretreated with BrfA, EBV-specific memory T-cell responses were significantly reduced by 1.6-fold in response to AC^EBV^-loaded B-LCLs (w/o: 18 spw; 5 µg/BrfA: 114 spw; **Figure 4C**). For unloaded B-LCLs, there was a slight yet not significant 1.2-fold reduction of T-cell responses (w/o: 168 spw; 5 µg/ml BrfA: 135 spw). In comparison to DMA and CCD, treatment with BrfA resulted in the smallest differences between AC^EBV^-loaded and unloaded B-LCLs. Of note, the expression of HLA class I on CD19^+^ and CD20^+^ B-LCLs was slightly downregulated upon addition of 0.1 µg/ml BrfA with no further decrease at higher concentrations (**Figure 4D**). In summary, the results of these *in vitro* inhibition assays suggest that EBV antigens present in AC^EBV^ can be cross-presented by B-LCLs, resulting in specific activation of EBV-specific memory T cells.

### AC^EBV^-mediated long-term proliferation of EBV-specific memory T cells

**Figure 5:**
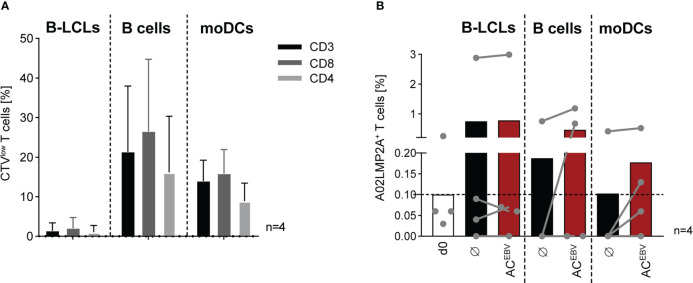
AC^EBV^-mediated long-term proliferation of EBV-specific memory T cells. Frequency of **(A)** CTV^low^ T cells (CD3, CD8, CD4) examined after seven days of stimulation with APCs loaded with the EBV antigen cocktail (AC^EBV^, E:T 10:1) and visualized after subtraction from those stimulated with unloaded APCs. Results of four independent experiments are expressed as mean ± SD. **(B)** Frequency of A02LMP2A^+^ T cells in HLA-A*02:01^+^, EBV-seropositive donors (n=4, grey dots) determined by A02LMP2A dextramer staining before and after stimulation (day 0/7). As control, unloaded APCs were co-cultured with autologous CD3^+^ T cells (Ø). Frequencies measured using a nonsense dextramer (background control) were subtracted. Results of four independent experiments are displayed as contiguous grey dots for individual donors and as mean values in response to unloaded and AC^EBV^-loaded. Statistical analysis was performed using the non-parametric Wilcoxon test. CTV, CellTrace Violet.

As an adjunct to short-term stimulation (**Figure 3**), the proliferative capacity of CTV-labeled EBV-specific memory T cells was assessed after seven day *in vitro* stimulation assays using autologous AC^EBV^-loaded APCs. Stimulation of T cells from seropositive donors with AC^EBV^-loaded B cells and DCs resulted in the highest T-cell proliferation (**Figure 5A**). In accordance with data shown in **Figures 3**, **4**, EBV-specific memory T-cell responses were always observed after stimulation with unloaded B-LCL and only slightly increased upon stimulation with AC^EBV^-loaded B-LCLs. Overall, the mean frequency of proliferating cells was slightly higher within CD8^+^ T cells compared to CD4^+^ T cells.

In addition, A02LMP2A multimer staining was performed to examine the frequency of EBV peptide-specific CD8^+^ T cells from HLA-A*02:01-positive donors in response to the AC^EBV^ (**Figure 5B**). Frequency of A02LMP2A^+^ T cells, which was 0.1% on day 0, increased after seven days of stimulation. Among the individual donors, the highest increase was observed after stimulation with AC^EBV^-loaded B cells and moDCs compared to stimulation with unloaded cells. Stimulation with B-LCLs resulted in the highest detected frequency of A02LMP2A^+^ T cells on average, but with only a slight difference between AC^EBV^-loaded and unloaded B-LCLs stimulation. In summary, these data show that EBV-specific T cells recognizing HLA-restricted epitopes proliferate in response to AC^EBV^.

### AC^EBV^ cross-presentation promotes EBV-specific memory T cells with high cytotoxic potential

**Figure 6:**
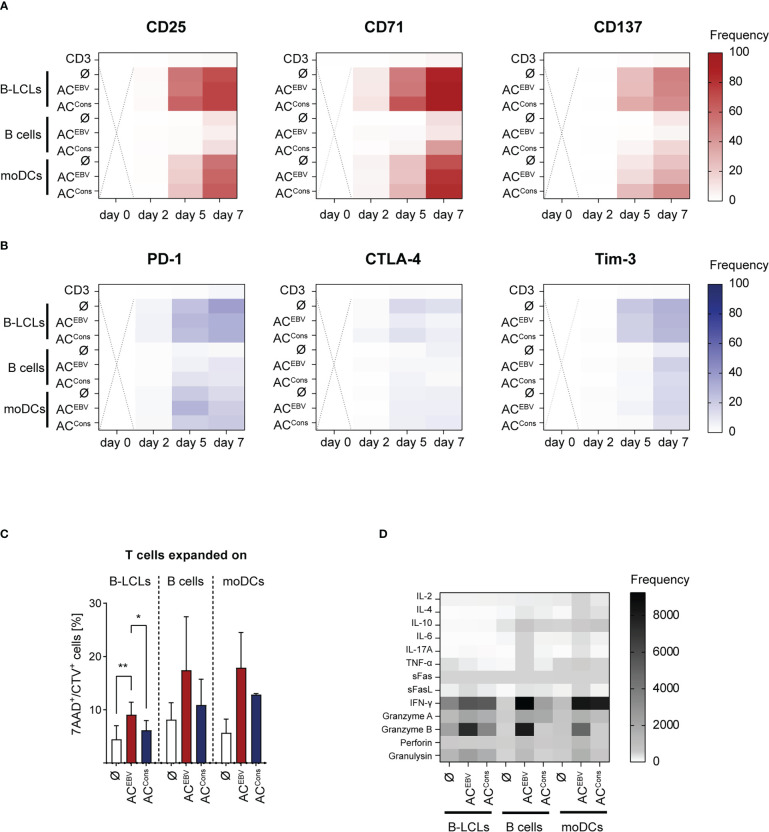
Characterization of EBV-specific memory T cells stimulated by AC^EBV^ cross-presentation. Heat maps display the expression of **(A)** activation maker and **(B)** exhaustion marker on CD3^+^ T cells from EBV-seropositive donors stimulated with AC^EBV^-loaded APCs on different days (day 0-7, n=4). CD3^+^ T cells alone and T-cell co-cultures with unloaded (Ø) or AC^Cons^-loaded APCs served as controls. The values are represented by different colors, as referenced in the bar. Statistical analysis was performed using the 2way ANOVA test followed by multiple comparison (ns - not significant). Dotted cross = not determined. **(C)** EBV-specific T cells expanded on AC-loaded (AC^EBV^, AC^Cons^) or unloaded APCs (Ø) for seven days were subjected to cytotoxicity assays using autologous unloaded B-LCLs as target cells in an effector to target ratio of 5:1. Frequencies of 7-AAD^+^ cells among CTV^+^ target cells (7AAD^+^/CTV^+^) are shown. PBMCs or B cells served as negative target cells and values obtained from these co-cultures were subtracted. Results of independent experiments (B-LCLs and B cells: n=3, moDC: n=2) are displayed as mean ± SD. Statistical analysis was performed using the non-parametric Wilcoxon test (*p< 0.05; **p< 0.01). **(D)** Cell culture supernatants from cytotoxicity assays from day eight were analyzed with respect to presence of cytotoxic effector molecules by LEGENDPlex Assay. (B-LCLs and B cells: n=3, moDC: n=2). The values are represented by different colors, as referenced in the bar. Statistical analysis was performed using the 2way ANOVA test followed by multiple comparison. AC^EBV^ - EBV antigen-containing cocktail, AC^Cons^ - EBV Consensus peptide pool.

Memory phenotype and functionality of CD3^+^ T cells stimulated over seven days with autologous AC^EBV^-loaded APCs were further characterized. APCs loaded with AC^Cons^ served as a control for EBV-specific T-cell activation. Continuous increase in T-cell activation, defined by the expression of CD25, CD71 and CD137, was observed in response to AC-loaded APCs. The strongest activation of CD3^+^ T cells was observed in response to AC^EBV^-loaded B-LCLs (CD25: 0.3% to 63.4%; CD71: 6.8% to 73.4%; CD137: 0.1% to 47.8%), which was comparable to AC^Cons^-loaded B-LCLs (**Figure 6A**). Of note, both CD8^+^ and CD4^+^ T cells were activated, with a tendency towards stronger activation of CD8^+^ T cells (**Supplemental Figure 2A**). These results suggest that antigens from AC^EBV^ mainly activate CD8^+^ T cells by cross-presentation *via* MHC class I. However, these antigens can also be processed into longer peptides and presented to CD4^+^ T cells *via* MHC class II. Furthermore, increased expression of the regulatory molecules PD-1 and Tim-3 on CD3^+^ T cells was observed on day 5 in all stimulation settings, while CTLA-4 expression increase only slightly (**Figure 6B**). The highest expression was detected for stimulation with AC^EBV^-loaded B-LCLs (PD-1: 4.3% to 35.7%; CTLA-4: 0.9% to 13.4%; Tim-3: 0.4% to 17.9%), with no significant differences between loaded or unloaded APCs, indicating that expression of all three markers on EBV-specific T cells is not directly affected in response to antigens from AC^EBV^. Similar to expression of activation marker, higher expression of the regulatory molecules was observed for CD8^+^ T cells compared to CD4^+^ T cells (**Supplemental Figure 2B**).

Additionally, the potential of EBV-specific T cells expanded on AC-loaded (AC^EBV^, AC^Cons^) or unloaded APCs to recognize and kill EBV antigen-presenting target cells was examined in *in vitro* cytotoxicity assay using autologous CTV-labeled B-LCLs as target cells in an E:T ratio of 5:1. Values obtained from co-cultures with CTV-labeled negative target cells (PBMCs or B cells) were subtracted. After four hours of co-culture, significantly increased frequencies of dead (7-AAD^+^) CTV^+^ target cells were observed in co-cultures with EBV-specific memory T cells expanded in presence of AC^EBV^ or AC^Cons^ when compared with T cells expanded in presence of unloaded APCs (**Figure 6C**). This effect was more pronounced in T cells expanded in presence of moDCs and B cells, although not reaching statistical significance. The ability of EBV-specific T cells to produce and secrete cytotoxic mediators during target cell recognition was examined in supernatants of above described co-cultures (**Figure 6D**; **Supplemental Figure 3**). Again, the cytotoxic potential of activated AC^EBV^- and AC^Cons^-specific T cells was evident in higher levels of the effector molecules like IFN-γ, granzyme B, perforin and granulysin compared with T cells stimulated with unloaded APCs. Overall, cross-presentation of AC^EBV^-derived antigens leads to activation of CD8^+^ T cells. The concomitant activation of CD4^+^ T cells suggests that AC^EBV^ antigen processing occurs also *via* the natural MHC class II pathway. Furthermore, cross-presentation of AC^EBV^-derived antigens efficiently induces functionally active EBV-specific memory T cells that are able to specifically recognize and kill EBV^+^ target cells and secrete cytotoxic effector molecules upon target recognition.

### Long-term stimulation with AC^EBV^-loaded B-LCLs leads to expansion of cytotoxic functional EBV-specific effector memory T cells

**Figure 7:**
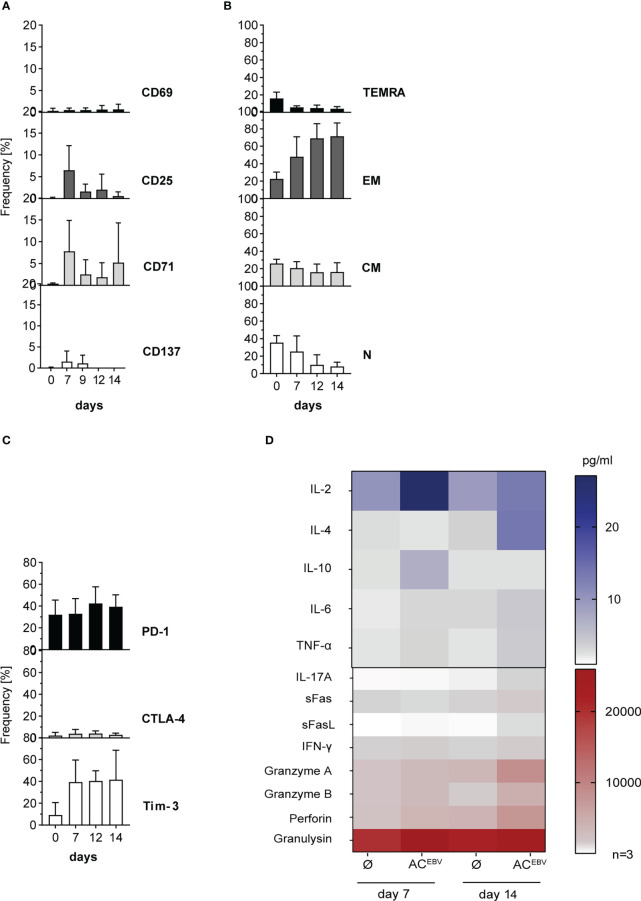
Phenotypic and functional characterization of EBV-specific memory T cells after long-term stimulation with AC^EBV^-loaded B-LCLs. CD3^+^ T cells characterized for **(A)** activation, **(B)** memory phenotype composition (N - naïve, CM - central memory. EM - effector memory, TEMRA - effector memory RA), and **(C)** exhaustion after long-term stimulation with autologous B-LCLs from EBV-seropositive donors loaded with the EBV antigen cocktail (AC^EBV^) on different days (day 0/7-14). For activation markers, the background of cells stimulated with unloaded B-LCLs (control) was subtracted from the corresponding sample, while for the exhaustion marker, a FMO (fluorescence minus one) was used as a control and subtracted for the corresponding sample. Results of three independent experiments are expressed as mean ± SD. Statistical analysis was performed using the 2way ANOVA test followed by multiple comparison. **(D)** Cell culture supernatants from 7 and 14 day T-cell stimulation assays were analyzed with respect to presence of cytotoxic effector molecules by LEGENDPlex Assay (n=3) and are displayed as heat maps. The values are represented by different colors, as referenced in the bar. Statistical analysis was performed using the 2way ANOVA test followed by multiple comparison.

Effects of long-term stimulation with antigens derived from rituximab-treated B-LCLs was investigated by restimulation with AC^EBV^-loaded and unloaded B-LCLs. Expression of all activation markers on CD3^+^ T cells decreased or remained unchanged after restimulation (day 7/14, CD137: 1.6%/0.0%; CD69: 0.5%/0.7%; CD25: 6.5%/0.6%; CD71: 7.8%/5.2%), with slightly higher expression on CD4^+^ T cells compared to CD8^+^ T cells. Only CD71, also identified as proliferation marker (33, 34), slightly increased from day 12 to day 14, but with a lower expression level compared to day 7 (**Figure 7A**; **Supplemental Figure 4**). Expanded EBV-specific CD3^+^ T cells mainly shifted towards effector memory phenotype (48.1% to 71.5%) as determined by CD45RA and CD62L expression (T_EM_: CD45RA^-^/CD62L^+^, **Figure 7B**). For CD8^+^ and CD4^+^ T cells similar tendency in CD45RA and CD62L expression were obtained. In addition, PD-1, CTLA-4 and Tim-3 expression on long-term stimulated EBV-specific CD3^+^ and CD8^+^ T cells remained unchanged, while expression of PD-1 and Tim-3 on CD4^+^ T cells was upregulated on day 12 and remained high (**Figure 7C**; **Supplemental Figures 4C, F**). The cytotoxic capacity of EBV-specific memory T cells was determined by secretion levels of CD8^+^ T cell-associated cytotoxic mediators in supernatants on days 7 and 14 of stimulation (**Figure 7D**). Highest secretion level were obtained for granulysin, followed by perforin, granzyme A, granzyme B, and IFN-γ after 14 days. Altogether, long-term stimulation with AC^EBV^-loaded B-LCLs leads to the expansion of EBV-specific memory T cells in which CD8^+^ T_EM_ cells appear to respond more rapidly when compared to CD4^+^ T_EM_ cells. Secretion of cytotoxic effector molecules was not affected by PD-1 and Tim-3 upregulation, suggesting that expanded EBV-specific memory T cells maintained their cytotoxic capacity.

## Discussion

The development of EBV-associated lymphomas is a consequence of the imbalance between immunosurveillance and immunosuppression and is often associated with inadequate EBV-specific T-cell responses in immunocompromised patients. Our previous study demonstrated a significant increase of EBV-specific T cells in pediatric patients during successful treatment of PTLD with rituximab (35). Based on these observations, the purpose of this study was to investigate whether treatment with rituximab can lead to restoration of cellular immunity by boosting the EBV-specific memory T-cell response through cross-presentation of antigens from rituximab-lysed EBV-infected B cells. Here we showed for the first time that rituximab-mediated lysis of EBV-infected cells can efficiently enhance activation and proliferation of EBV-specific CD8^+^ CTLs of effector memory T-cell phenotype (T_EM_) through cross-presentation of EBV-derived antigens by DCs, B cells as well as by B-LCLs *via* MHC class I molecules. This promotes the restoration of antiviral cellular immunity.

### B-LCLs are capable to cross-present exogenous antigens in a TAP-dependent way

Uptake of extracellular antigens occurs mainly by receptor-mediated endocytosis, phagocytosis, and macropinocytosis and appears to be different among APC subtypes due to the variety of receptors involved (e.g., B cell receptor, heat shock protein receptors, Fc receptors) (36). Consistent with previous observations, TAP-dependent peptide translocation in B-LCLs was comparable to that of monocytes and B cells (30, 32). As professional APCs, DCs are able to present exogenous antigens with higher efficiency when compared to B cells, monocytes, and macrophages (36). Therefore, higher cell numbers and E:T ratios were required for *in vitro* stimulation experiments with B cells and B-LCLs to achieve T-cell responses comparable to those in moDCs. Exogenous antigens are cross-presented on these cell types with shorter residence time in the peptide/MHC class I complexes on the cell surface as compared to moDCs (32). The rapid exchange of peptide/MHC class I complexes on B cells, monocytes, and B-LCLs could result in the appropriate antigen-specific T-cell responses being triggered with low efficiency or not at all, but this could not be confirmed by previous analyses. Moreover, DCs have been shown to be able to present antigens from apoptotic, untreated and rituximab-treated lymphoma cells, with endocytosis of rituximab-treated malignant B cells being most pronounced (37). Until now, the role of B cells in the induction of CD8^+^ T cells by cross-presentation has been largely unclear, but our results suggest a pivotal role. Strong reduction of EBV-specific T-cells responses due to the inhibition of antigen uptake in both, AC^EBV^-loaded and unloaded B-LCLs proposes that a large proportion of the immunogenic antigens presented by B-LCLs appear to be exogenous and originate from surrounding apoptotic and untreated B-LCLs.

### Rituximab-mediated activation of reactive EBV-specific CD8^+^ and CD4^+^ memory T cells, with CD8^+^ T cells are more effective

Our previous observations of patients in whom we detected increased EBV-specific T-cell responses *in vivo* during reduction of immunosuppression and with rituximab treatment (35) were confirmed by *in vitro* immunoassays performed in this study. Activation of EBV-specific memory T cells was clearly demonstrated generally and also specifically by increased frequency of A02LMP2A^+^ T cells after AC^EBV^-dependent stimulation. EBV-specific memory T-cell responses were significantly boosted by AC^EBV^ antigen cross-presentation, independent of bystander T-cell activation by B-LCLs as a consequence of the expression of EBV proteins associated with latency states II and III. Once T cells are activated, regulatory proteins such as PD-1, CTLA-4, and Tim-3 are expressed. On the other hand, continuously high expression of these inhibitory receptors is associated with T-cell exhaustion (38). In this study, despite the upregulation of PD-1 and Tim-3 but not CTLA-4 on CD8^+^ and CD4^+^ T cells, proliferative capacity and cytotoxic effector function were not impaired throughout the course of stimulation, suggesting that EBV-specific memory T cells were activated but not exhausted. Cross-presentation of AC^EBV^-derived antigens leads to a stronger activation and proliferation of CD8^+^ memory T cells than CD4^+^ memory T cells at early time points, suggesting that the AC^EBV^ mainly processed to peptides of 8-12 amino acids in length, which arepresented *via* MHC class I molecules (39, 40). Since activation of CD4^+^ T cells also occurs, it can be assumed that AC^EBV^-antigen processing occurs also *via* the natural MHC class II pathway (41). In context to EBV-specific CD4^+^ T cells responses, our data are consistent with previous studies in patients with EBV-associated PTLD, showing considerably increased CD8^+^ T-cell responses but only a moderate increase of CD4^+^ T-cell responses after reduction in immunosuppression and rituximab treatment (35). One possible explanation could be immune evasion by EBV, encoding for a number of evasion molecules, including proteins and miRNAs, that are able to directly interfere with and reduce antigen presentation of EBV-infected cells (42). Most EBV evasion proteins involved in downregulation of MHC class II are the lytic proteins BZLF2, BGLF5, BCRF1, and BDLF3. Disrupted MHC class II regulation could be triggered by latent proteins such as EBNA2, which could explain the observed moderate and delayed CD4^+^ T-cell responses.

### Impact of the tumor environment on rituximab-mediated T-cell responses

Enhancement of EBV-specific T-cell responses due to the release of EBV antigens after rituximab-mediated cell lysis cannot be observed in all patients (35). There is increasing evidence that EBV-infected tumor cells can influence the tumor microenvironment to their own advantage by creating an immunosuppressive environment. Studies of solid and hematological EBV-associated malignancies have shown that immunosuppressive IL-10 levels are increased when compared with EBV-negative malignancies, leading to the promotion of an inhibitory or tolerogenic immune environment (42). Furthermore, migration of IL-10-secreting regulatory CD4^+^ T cells (Tregs) into the tumor environment can be induced by EBNA1-mediated upregulation of CCL20 on tumor cells, leading to the induction of immune tolerance through the suppression of cytotoxic CD8^+^ T cells (43, 44). However, EBV-specific CD8^+^ T-cell responses analyzed in this study were not suppressed by IL-10 since no significant changes in IL-10 secretion in co-cultures of CD3^+^ T cells with loaded or unloaded target cells were observed. In addition to the direct effect of AC^EBV^ cross-presentation on the induction of EBV-specific memory T cells investigated here, the influence of the tumor microenvironment and the interaction with other immune cells has to be addressed in future studies.

### The release of EBV antigens is conceivable for other therapeutic antibodies or even for therapeutically applied T cells

Treatment with rituximab specifically eliminates CD20^+^ B cells without inhibiting antibody production by CD138^+^ plasma cells. Regeneration of B cells from stem cells and pro-B lymphocytes is also not affected (45). Usually, rituximab is a well-tolerated biologic agent and severe side effects such as cytokine release syndrome (CRS) and acute tumor lysis syndrome (ATLS) are rare (46, 47). The usage of other anti-CD20 antibodies (e.g., obinutuzumab, ofatumumab, veltuzumab, and ocrelizumab) in rituximab-resistant patients should also lead to cross-presentation of EBV antigens. In rituximab-resistant patients, EBV^+^ lymphomas have been treated with other anti-CD20 antibodies (e.g., obinutuzumab, ofatumumab, veltuzumab, and ocrelizumab) or with the CD30-directed antibody drug conjugate (ADC) brentuximab vedotin. It is likely that lysis by brentuximab vedotin might also result in the release of EBV antigens and therefore boost the EBV-specific memory T-cell responses against residual tumor cells. In an individual patient treatment, we have demonstrated successful treatment of chemotherapy-refractory EBV^+^ PTLD in a patient after HSCT by a combination of brentuximab-vedotin and transfer of EBV-specific T cells (48). Of note, CD30 is expressed at low levels on activated antiviral memory T cells, potentially making them a target of brentuximab vedotin (49). Subsequent *in vitro* studies should examine this hypothesis to further elucidate the mechanisms of antibody-mediated lysis of infected cells and the role of cellular immunity in their elimination. During treatment with rituximab, reduction of immunosuppression may result in more effective activation of EBV-specific memory T cells through cross-presentation of released EBV antigens. Combination of rituximab administration and EBV-specific T cells might improve treatment efficacy and build robust antitumor immunity with longer recurrence-free survival by three parallel mechanisms: depletion of malignant EBV^+^ B cells, suppression of general inflammation, and supported long-term reconstitution of T-cell immunity. In a prospective study, an increase in the complete remission rates of 95% in patients with EBV-associated PTLD was shown by rituximab administration following adoptive transfer of DLIs or autologous EBV-CTLs resulting in a five-year overall survival in 71% of patients (50). In this context, our data indicate that a 4^th^ mechanism of action of rituximab-mediated EBV-specific T-cell immunity may also be possible here, namely boosting of EBV-specific T-cell responses by the release of EBV antigens from infected cells by (i) antibody-mediated lysis or by the (ii) cytotoxic activity of the transferred and patient’s own endogenous T cells. In addition, it can be hypothesized that rituximab treatment and adoptive transfer of genetically engineered T cells (51, 52) (53) may also result in a massive release of EBV antigens and subsequent boosting of specific T-cell responses.

## Conclusion

Our data support the hypothesis that cross-presentation of antigens derived from rituximab-treated EBV-infected cells can enhance EBV-specific memory T-cell responses and likely then lead to effective elimination of residual EBV-infected malignant B cells *in vivo*. Thus, the enhancement of cell-mediated immunity by EBV-specific T cells is novel efficient mechanism of targeted rituximab therapy for the treatment of CD20^+^ EBV-associated lymphomas, in addition to antibody-mediated lysis. EBV provides a well-characterized surrogate antigen in our system. However, it can be hypothesized that through this mechanism of action, T cells specific to other tumor-associated antigens might also expand in CD20^+^ lymphomas without EBV and thus contribute to tumor-directed immunity and elimination of malignant cells. Challenges based in the tumor microenvironment and tumor evasion strategies should be considered in further studies on the mechanism of action and the development of future therapies.

## Data availability statement

The original contributions presented in the study are included in the article/**Supplementary Material**. Further inquiries can be directed to the corresponding author.

## Ethics statement

The studies involving human participants were reviewed and approved by Hannover Medical School, Ethics Committee. The patients/participants provided their written informed consent to participate in this study.

## Author contributions

ST-Z - Data acquisition, analysis, writing, final approval; AB - Data acquisition, analysis, writing, final approval; please include for MS, PM and SS final approval; MD – performance of the TAP assay, analysis, final approval; RB - final approval; UK - final approval; BM-K - Study design, clinical background, final approval; BE-V - Study design, analysis, writing, final approval.
