# Supplementary material for: Reinforcement of cell-mediated immunity driven by tumor-associated Epstein-Barr virus (EBV)-specific T cells during targeted B-cell therapy with rituximab
Source: Front Immunol. 2023 Mar 24;14:878953. doi: 10.3389/fimmu.2023.878953 (PMC10079996; doi:10.3389/fimmu.2023.878953)
Supplement: Supplementary file 1 [file DataSheet_1.pdf]

Supplementary Material

Supplemental Figures

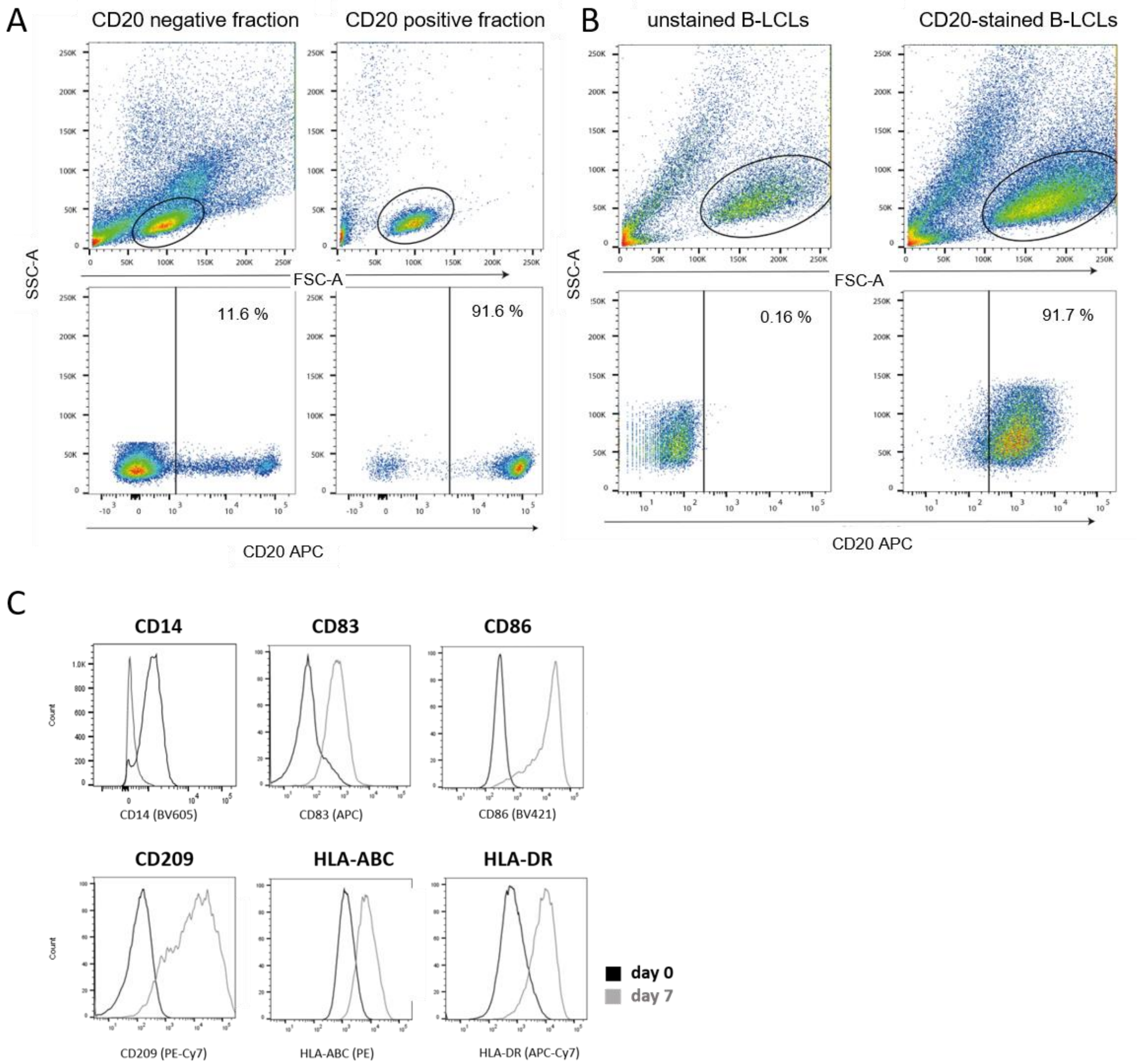

Supplemental Figure 1: Flow cytometric analysis of B cells, B-LCLs and moDCs

Frequency of **(A)** $CD20^+$ B cells in the negative and positive fraction after $CD20^+$ B-cell isolation and frequency of **(B)** CD20-expressing EBV-immortalized B-lymphoblastoid cell lines (B-LCLs). **(C)** Expression of CD14, CD83, CD86, CD209, HLA ABC and HLA-DR during differentiation from monocytes (day 0, open black line histograms) to mature monocyte-derived dendritic cells (moDCs, day 7 open grey line histograms). All dot plots and histograms are representative of one independent experiment.

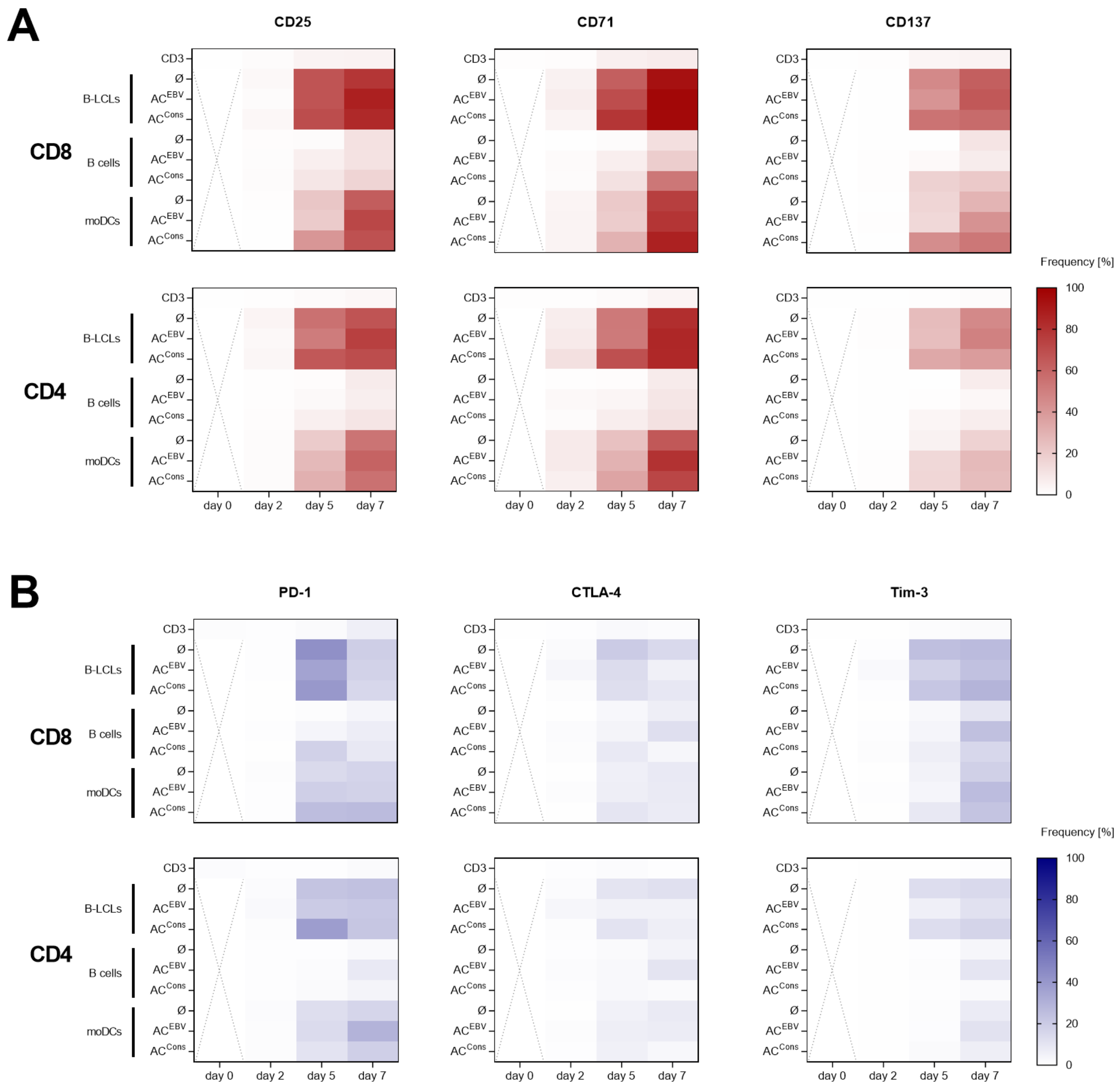

Supplemental Figure 2: Characterization of EBV-specific T cells stimulated with AC^{EBV}-loaded APCs.

Heat maps display the expression of **(A)** activation maker and **(B)** exhaustion marker on CD8^{+} and CD4^{+} T cells from EBV-seropositive donors stimulated with AC^{EBV}-loaded APCs on different days (day 0-7, n=4). CD3^{+} T cells alone and T-cell co-cultures with unloaded (Ø) or AC^{Cons}-loaded APCs served as controls. The values are represented by different colors, as referenced in the bar. Statistical analysis was performed using the 2way ANOVA test followed by multiple comparison. Dotted cross = not determined.

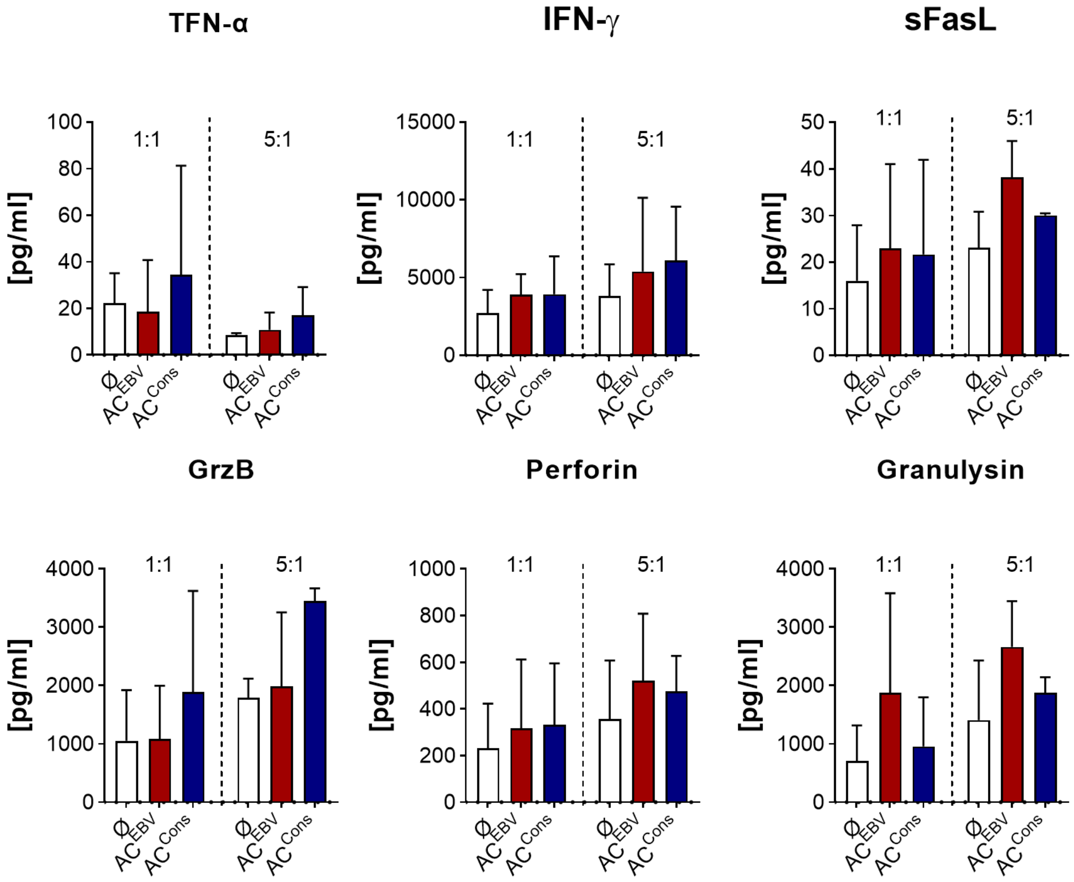

Supplemental Figure 3: Cytotoxic potential of EBV-specific T cells stimulated with AC^{EBV}-loaded B-LCLs

EBV-specific T cells expanded on AC-loaded (AC^{EBV}, AC^{Cons}) or unloaded B-LCLs (Ø) for seven days were subjected to cytotoxicity assays using autologous unloaded B-LCLs as target cells in an effector to target ratio of 1:1 and 5:1. Cell culture supernatants from cytotoxicity assays from day eight were analyzed with respect to presence of cytotoxic effector molecules by LEGENDPlex Assay. Results of three independent experiments are expressed as mean ± SD. Statistical analysis was performed using the non-parametric Wilcoxon test. AC^{EBV} - EBV antigen-containing cocktail, AC^{Cons} - EBV Consensus peptide pool.

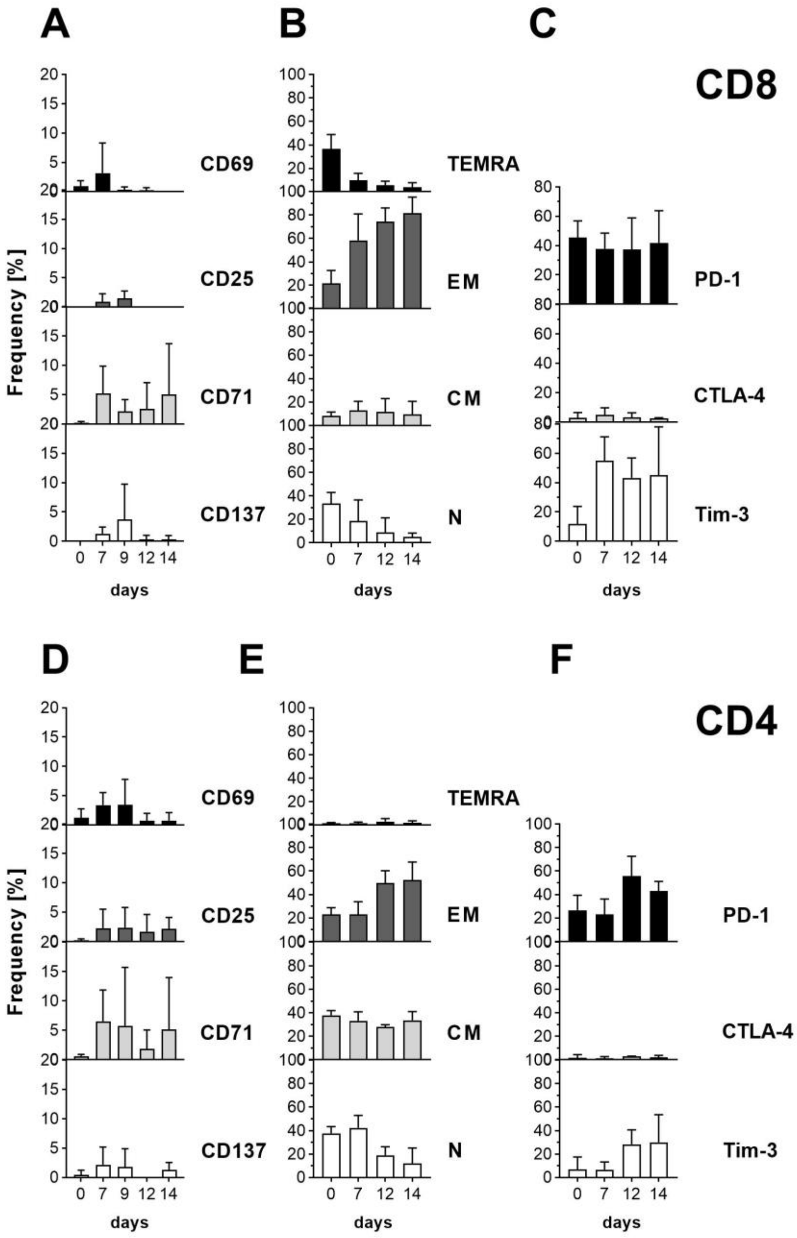

Supplementary Figure 4: Phenotypic characterization of EBV-specific memory T cells after long-term stimulation with AC^{EBV}-loaded B-LCLs.

$CD8^+$ and CD4+ T cells characterized for **(A, D)** activation, **(B, E)** memory phenotype composition (N - naïve, CM - central memory. EM - effector memory, TEMRA - effector memory RA), and **(C, F)** exhaustion after long-term stimulation with autologous B-LCLs from EBV-seropositive donors loaded with the EBV antigen cocktail (AC^{EBV}) on different days (day 0/7-14). For activation markers, the background of cells stimulated with unloaded B-LCLs (control) was subtracted from the corresponding sample, while for the exhaustion marker, a FMO (fluorescence minus one) was used as a control and subtracted for the corresponding sample. Results of three independent experiments are expressed as mean ± SD. Statistical analysis was performed using the 2way ANOVA test followed by multiple comparison.
